# Supplementary material for: Emergence and evolution of an interaction between intrinsically disordered proteins
Source: eLife. 2017 Apr 11;6:e16059. doi: 10.7554/eLife.16059 (PMC5419745; doi:10.7554/eLife.16059)
Supplement: Figure 2—source data 1. — DOI: http://dx.doi.org/10.7554/eLife.16059.005 [file elife-16059-fig2-data1.docx]

**Figure 2 – source data 1.** Probabilities of resurrected amino acid residues at the respective position (2062-2109) in the NCBD domain.

>Node NCBD CREBBP teleost fish/tetrapod

A C D E F G H I K L M N P Q R S T V W Y

2062 0 0 0 0 0 0 0 1 0 0 0 0 0 0 0 0 0 0 0 0

2063 0.02 0 0 0 0 0 0 0 0 0 0 0 0.91 0 0 0.06 0 0 0 0

2064 0 0 0 0 0 0 0 0 0 0 0 0 1 0 0 0 0 0 0 0

2065 0 0 0 0 0 0 0 0 0 0 0 1 0 0 0 0 0 0 0 0

2066 1 0 0 0 0 0 0 0 0 0 0 0 0 0 0 0 0 0 0 0

2067 0 0 0 0 0 0 0 0 0 1 0 0 0 0 0 0 0 0 0 0

2068 0 0 0 0 0 0 0 0 0 0 0 0 0 1 0 0 0 0 0 0

2069 0 0 1 0 0 0 0 0 0 0 0 0 0 0 0 0 0 0 0 0

2070 0 0 0 0 0 0 0 0 0 1 0 0 0 0 0 0 0 0 0 0

2071 0 0 0 0 0 0 0 0 0 1 0 0 0 0 0 0 0 0 0 0

2072 0 0 0 0 0 0 0 0 0 0 0 0 0 0 1 0 0 0 0 0

2073 0 0 0 0 0 0 0 0 0 0 0 0 0 0 0 0 1 0 0 0

2074 0 0 0 0 0 0 0 0 0 1 0 0 0 0 0 0 0 0 0 0

2075 0 0 0 0 0 0 0 0 1 0 0 0 0 0 0 0 0 0 0 0

GAP 0 0 0 0 0 0 0 0 0 0 0 0 0 0 0 0 0 0 0 0

2076 0 0 0 0 0 0 0 0 0 0 0 0 0 0 0 1 0 0 0 0

2077 0 0 0 0 0 0 0 0 0 0 0 0 1 0 0 0 0 0 0 0

2078 0 0 0 0 0 0 0 0 0 0 0 0 0 0 0 1 0 0 0 0

GAP 0 0 0 0 0 0 0 0 0 0 0 0 0 0 0 0 0 0 0 0

2079 0 0 0 0 0 0 0 0 0 0 0 0 0 0 0 1 0 0 0 0

GAP 0 0 0 0 0 0 0 0 0 0 0 0 0 0 0 0 0 0 0 0

2080 0 0 0 0 0 0 0 0 0 0 0 0 1 0 0 0 0 0 0 0

2081 0 0 0 0 0 0 0 0 0 0 0 0 0 1 0 0 0 0 0 0

2082 0 0 0 0 0 0 0 0 0 0 0 0 0 1 0 0 0 0 0 0

2083 0 0 0 0 0 0 0 0 0 0 0 0 0 1 0 0 0 0 0 0

2084 0 0 0 0 0 0 0 0 0 0 0 0 0 1 0 0 0 0 0 0

2085 0 0 0 0 0 0 0 0 0 0 0 0 0 1 0 0 0 0 0 0

GAP 0 0 0 0 0 0 0 0 0 0 0 0 0 0 0 0 0 0 0 0

2086 0 0 0 0 0 0 0 0 0 0 0 0 0 0 0 0 0 1 0 0

2087 0 0 0 0 0 0 0 0 0 1 0 0 0 0 0 0 0 0 0 0

2088 0 0 0 0 0 0 0 0 0 0 0 1 0 0 0 0 0 0 0 0

2089 0 0 0 0 0 0 0 1 0 0 0 0 0 0 0 0 0 0 0 0

2090 0 0 0 0 0 0 0 0 0 1 0 0 0 0 0 0 0 0 0 0

2091 0 0 0 0 0 0 0 0 1 0 0 0 0 0 0 0 0 0 0 0

2092 0 0 0 0 0 0 0 0 0 0 0 0 0 0 0 1 0 0 0 0

2093 0 0 0 0 0 0 0 0 0 0 0 1 0 0 0 0 0 0 0 0

2094 0 0 0 0 0 0 0 0 0 0 0 0 1 0 0 0 0 0 0 0

2095 0 0 0 0 0 0 0 0 0 0 0 0 0 1 0 0 0 0 0 0

2096 0 0 0 0 0 0 0 0 0 1 0 0 0 0 0 0 0 0 0 0

2097 0 0 0 0 0 0 0 0 0 0 1 0 0 0 0 0 0 0 0 0

2098 1 0 0 0 0 0 0 0 0 0 0 0 0 0 0 0 0 0 0 0

2099 1 0 0 0 0 0 0 0 0 0 0 0 0 0 0 0 0 0 0 0

2100 0 0 0 0 1 0 0 0 0 0 0 0 0 0 0 0 0 0 0 0

2101 0 0 0 0 0 0 0 1 0 0 0 0 0 0 0 0 0 0 0 0

2102 0 0 0 0 0 0 0 0 1 0 0 0 0 0 0 0 0 0 0 0

2103 0 0 0 0 0 0 0 0 0 0 0 0 0 1 0 0 0 0 0 0

2104 0 0 0 0 0 0 0 0 0 0 0 0 0 0 1 0 0 0 0 0

2105 0 0 0 0 0 0 0 0 0 0 0 0 0 0 0 0 1 0 0 0

2106 1 0 0 0 0 0 0 0 0 0 0 0 0 0 0 0 0 0 0 0

2107 0 0 0 0 0 0 0 0 1 0 0 0 0 0 0 0 0 0 0 0

2108 0 0 0 0 0 0 0 0 0 0 0 0 0 0 0 0 0 0 0 1

2109 0 0 0 0 0 0 0 0 0 0 0 0 0 1 0 0 0 0 0 0

>Node NCBD 1R/2R

A C D E F G H I K L M N P Q R S T V W Y

2062 0 0 0 0 0 0 0 0.98 0 0.01 0 0 0 0 0 0 0 0.01 0 0

2063 0 0 0 0 0 0 0 0 0 0 0 0 1 0 0 0 0 0 0 0

2064 0 0 0 0 0 0 0 0 0 0 0 0 0.99 0.01 0 0 0 0 0 0

2065 0.05 0 0.01 0 0 0.08 0.01 0 0 0 0 0.52 0 0.01 0 0.3 0.02 0 0 0

2066 1 0 0 0 0 0 0 0 0 0 0 0 0 0 0 0 0 0 0 0

2067 0 0 0 0 0 0 0 0 0 1 0 0 0 0 0 0 0 0 0 0

2068 0 0 0 0 0 0 0 0 0 0 0 0 0 1 0 0 0 0 0 0

2069 0 0 1 0 0 0 0 0 0 0 0 0 0 0 0 0 0 0 0 0

2070 0 0 0 0 0 0 0 0 0 1 0 0 0 0 0 0 0 0 0 0

2071 0 0 0 0 0 0 0 0 0 1 0 0 0 0 0 0 0 0 0 0

2072 0 0 0 0 0 0 0 0 0 0 0 0 0 0 1 0 0 0 0 0

2073 0 0 0 0 0 0 0 0 0 0 0 0 0 0 0 0 1 0 0 0

2074 0 0 0 0 0 0 0 0 0 1 0 0 0 0 0 0 0 0 0 0

2075 0 0 0 0 0 0 0 0 0.27 0 0 0 0 0 0.73 0 0 0 0 0

GAP 0 0 0 0 0 0 0 0 0 0 0 0 0 0 0 0 0 0 0 0

2076 0 0 0 0 0 0 0 0 0 0 0 0 0 0 0 1 0 0 0 0

2077 0 0 0 0 0 0 0 0 0 0 0 0 1 0 0 0 0 0 0 0

2078 0 0 0 0 0 0 0 0 0 0 0 0 0 0 0 1 0 0 0 0

GAP 0 0 0 0 0 0 0 0 0 0 0 0 0 0 0 0 0 0 0 0

2079 0 0 0 0 0 0 0 0 0 0 0 0 0 0 0 1 0 0 0 0

GAP 0 0 0 0 0 0 0 0 0 0 0 0 0 0 0 0 0 0 0 0

2080 0 0 0 0 0 0 0 0 0 0 0 0 1 0 0 0 0 0 0 0

2081 0 0 0 0 0 0 0 0 0 0 0 0 0 1 0 0 0 0 0 0

2082 0 0 0 0 0 0 0 0 0 0 0 0 0 1 0 0 0 0 0 0

2083 0 0 0 0 0 0 0 0 0 0 0 0 0 1 0 0 0 0 0 0

2084 0 0 0 0 0 0 0 0 0 0 0 0 0 1 0 0 0 0 0 0

2085 0 0 0 0 0 0 0 0 0 0 0 0 0 1 0 0 0 0 0 0

GAP 0 0 0 0 0 0 0 0 0 0 0 0 0 0 0 0 0 0 0 0

2086 0 0 0 0 0 0 0 0 0 0 0 0 0 0 0 0 0 1 0 0

2087 0 0 0 0 0 0 0 0 0 1 0 0 0 0 0 0 0 0 0 0

2088 0 0 0 0 0 0 0 0 0 0 0 1 0 0 0 0 0 0 0 0

2089 0 0 0 0 0 0 0 1 0 0 0 0 0 0 0 0 0 0 0 0

2090 0 0 0 0 0 0 0 0 0 1 0 0 0 0 0 0 0 0 0 0

2091 0 0 0 0 0 0 0 0 0.99 0 0 0 0 0 0.01 0 0 0 0 0

2092 0 0 0 0 0 0 0 0 0 0 0 0 0 0 0 1 0 0 0 0

2093 0 0 0 0 0 0 0 0 0 0 0 1 0 0 0 0 0 0 0 0

2094 0 0 0 0 0 0 0 0 0 0 0 0 1 0 0 0 0 0 0 0

2095 0 0 0 0 0 0 0 0 0 0 0 0 0 1 0 0 0 0 0 0

2096 0 0 0 0 0 0 0 0 0 1 0 0 0 0 0 0 0 0 0 0

2097 0 0 0 0 0 0 0 0 0 0 1 0 0 0 0 0 0 0 0 0

2098 1 0 0 0 0 0 0 0 0 0 0 0 0 0 0 0 0 0 0 0

2099 1 0 0 0 0 0 0 0 0 0 0 0 0 0 0 0 0 0 0 0

2100 0 0 0 0 1 0 0 0 0 0 0 0 0 0 0 0 0 0 0 0

2101 0 0 0 0 0 0 0 1 0 0 0 0 0 0 0 0 0 0 0 0

2102 0 0 0 0 0 0 0 0 1 0 0 0 0 0 0 0 0 0 0 0

2103 0 0 0 0 0 0 0 0 0 0 0 0 0 1 0 0 0 0 0 0

2104 0 0 0 0 0 0 0 0 0 0 0 0 0 0 1 0 0 0 0 0

2105 0.81 0 0 0 0 0 0 0 0 0 0 0 0 0 0 0.01 0.18 0 0 0

2106 0.98 0 0 0 0 0 0 0 0 0 0 0 0 0 0 0.01 0 0 0 0

2107 0 0 0 0 0 0 0 0 0.64 0 0 0 0 0 0.35 0 0 0 0 0

2108 0 0 0 0 0 0 0 0 0 0 0 0 0 0 0 0 0 0 0 1

2109 0 0 0 0 0 0 0 0 0 0 0 0 0 1 0 0 0 0 0 0

>Node NCBD Deuterostomes/Protostomes

A C D E F G H I K L M N P Q R S T V W Y

2062 0.07 0 0 0 0.06 0 0.01 0.19 0 0.05 0.13 0.02 0.06 0.01 0 0.07 0.15 0.16 0 0.01

2063 0.03 0 0 0 0 0 0 0.02 0 0.08 0.1 0 0.67 0 0 0.03 0.04 0.02 0 0

2064 0 0 0 0 0 0 0 0 0 0 0 0 1 0 0 0 0 0 0 0

2065 0 0 0 0 0 0 0.1 0 0 0 0 0 0.12 0.75 0.01 0 0 0 0 0

2066 1 0 0 0 0 0 0 0 0 0 0 0 0 0 0 0 0 0 0 0

2067 0 0 0 0 0 0 0 0 0 1 0 0 0 0 0 0 0 0 0 0

2068 0 0 0 0 0 0 0 0 0 0 0 0 0 1 0 0 0 0 0 0

2069 0 0 0 0 0 0 0 0 0 0 0 0 0 1 0 0 0 0 0 0

2070 0 0 0 0 0 0 0 0 0 1 0 0 0 0 0 0 0 0 0 0

2071 0 0 0 0 0 0 0 0 0 1 0 0 0 0 0 0 0 0 0 0

2072 0 0 0 0 0 0 0 0 0 0 0 0 0 1 0 0 0 0 0 0

2073 0 0 0 0 0 0 0 0 0 0 0 0 0 0 0 0 1 0 0 0

2074 0 0 0 0 0 0 0 0 0 1 0 0 0 0 0 0 0 0 0 0

2075 0 0 0 0 0 0 0 0 1 0 0 0 0 0 0 0 0 0 0 0

GAP 0 0 0 0 0 0 0 0 0 0 0 0 0 0 0 0 0 0 0 0

2076 0 0 0 0 0 0 0 0 0 0 0 0 0 0 0 1 0 0 0 0

2077 0 0 0 0 0 0 0 0 0 0 0 0 1 0 0 0 0 0 0 0

2078 0 0 0 0 0 0 0 0 0 0 0 0.09 0 0 0 0.91 0 0 0 0

GAP 0 0 0 0 0 0 0 0 0 0 0 0 0 0 0 0 0 0 0 0

2079 0 0 0 0 0 0 0 0 0 0 0 0 0 0 0 1 0 0 0 0

GAP 0 0 0 0 0 0 0 0 0 0 0 0 0 0 0 0 0 0 0 0

2080 0 0 0 0 0 0 0 0 0 0 0 0 1 0 0 0 0 0 0 0

2081 0 0 0 0 0 0 0 0 0 0 0 0 0 1 0 0 0 0 0 0

2082 0 0 0 0 0 0 0 0 0 0 0 0 0 1 0 0 0 0 0 0

2083 0 0 0 0 0 0 0 0 0 0 0 0 0 1 0 0 0 0 0 0

2084 0 0 0 0.01 0 0 0 0 0 0 0 0 0 0.98 0 0 0 0 0 0

2085 0 0 0 0 0 0 0 0 0 0 0 0 0 1 0 0 0 0 0 0

GAP 0 0 0 0 0 0 0 0 0 0 0 0 0 0 0 0 0 0 0 0

2086 0 0 0 0 0 0 0 0 0 0 0 0 0 0 0 0 0 1 0 0

2087 0 0 0 0 0 0 0 0 0 1 0 0 0 0 0 0 0 0 0 0

2088 0 0 0 0 0 0 0.34 0 0 0 0 0.26 0 0.37 0 0.01 0 0 0 0

2089 0 0 0 0 0 0 0 1 0 0 0 0 0 0 0 0 0 0 0 0

2090 0 0 0 0 0 0 0 0 0 1 0 0 0 0 0 0 0 0 0 0

2091 0 0 0 0 0 0 0 0 1 0 0 0 0 0 0 0 0 0 0 0

2092 0 0 0 0 0 0 0 0 0 0 0 0 0 0 0 1 0 0 0 0

2093 0 0 0 0 0 0 0 0 0 0 0 1 0 0 0 0 0 0 0 0

2094 0 0 0 0 0 0 0 0 0 0 0 0 1 0 0 0 0 0 0 0

2095 0 0 0 0 0 0 0 0 0 0 0 0 0 1 0 0 0 0 0 0

2096 0 0 0 0 0 0 0 0 0 1 0 0 0 0 0 0 0 0 0 0

2097 0 0 0 0 0 0 0 0 0 0 1 0 0 0 0 0 0 0 0 0

2098 1 0 0 0 0 0 0 0 0 0 0 0 0 0 0 0 0 0 0 0

2099 1 0 0 0 0 0 0 0 0 0 0 0 0 0 0 0 0 0 0 0

2100 0 0 0 0 1 0 0 0 0 0 0 0 0 0 0 0 0 0 0 0

2101 0 0 0 0 0 0 0 1 0 0 0 0 0 0 0 0 0 0 0 0

2102 0 0 0 0 0 0 0 0 1 0 0 0 0 0 0 0 0 0 0 0

2103 0 0 0 0 0 0 0 0 0 0 0 0 0 1 0 0 0 0 0 0

2104 0 0 0 0 0 0 0 0 0 0 0 0 0 0 1 0 0 0 0 0

2105 0.01 0 0 0 0 0 0.01 0 0 0 0 0.01 0 0 0 0.93 0.03 0 0 0

2106 0 0 0 0 0 0 0 0 0 0 0 0 0 1 0 0 0 0 0 0

2107 0 0 0 0 0 0 0.87 0 0 0 0 0 0 0.12 0.01 0 0 0 0 0

2108 0 0 0 0 0 0 0 0 0 0 0 0 0 1 0 0 0 0 0 0

2109 0 0 0 0 0 0 0 0 0 0 0 0 0 1 0 0 0 0 0 0
