# Supplementary material for: Emergence and evolution of an interaction between intrinsically disordered proteins
Source: eLife. 2017 Apr 11;6:e16059. doi: 10.7554/eLife.16059 (PMC5419745; doi:10.7554/eLife.16059)
Supplement: Figure 2—source data 2. — The gap in Figure 2—figure supplement 4 created by one of the Takifugu rubripes sequences was removed in this table to make it easier to understand the numbering. DOI: http://dx.doi.org/10.7554/eLife.16059.006 [file elife-16059-fig2-data2.docx]

**Figure 2 – source data 2.** Probabilities of resurrected amino acid residues at the respective position (1040-1081) in the CID domain. The gap in **Fig. 2 - figure supplement 4**created by one of the Takifugu rubripes sequences was removed in this table to make it easier to understand the numbering.

>Node CID3 teleost fish/tetrapod

A C D E F G H I K L M N P Q R S T V W Y

1040 0 0 0 1 0 0 0 0 0 0 0 0 0 0 0 0 0 0 0 0

1041 0 0 0 0 0 0.99 0 0 0 0 0 0 0 0 0 0 0 0 0 0

1042 0 0 0 0 0 0 0 0 0 0 0 0 0 1 0 0 0 0 0 0

1043 0 0 0 0 0 0 0 0 0 0 0 0.03 0 0 0 0.96 0 0 0 0

1044 0 0 1 0 0 0 0 0 0 0 0 0 0 0 0 0 0 0 0 0

1045 0 0 0 1 0 0 0 0 0 0 0 0 0 0 0 0 0 0 0 0

1046 0 0 0 0 0 0 0 0 0.01 0 0 0 0 0 0.99 0 0 0 0 0

1047 1 0 0 0 0 0 0 0 0 0 0 0 0 0 0 0 0 0 0 0

1048 0 0 0 0 0 0 0 0 0 1 0 0 0 0 0 0 0 0 0 0

1049 0 0 0 0 0 0 0 0 0 1 0 0 0 0 0 0 0 0 0 0

1050 0 0 1 0 0 0 0 0 0 0 0 0 0 0 0 0 0 0 0 0

1051 0 0 0 0 0 0 0 0 0 0 0 0 0 1 0 0 0 0 0 0

1052 0 0 0 0 0 0 0 0 0 1 0 0 0 0 0 0 0 0 0 0

1053 0 0 1 0 0 0 0 0 0 0 0 0 0 0 0 0 0 0 0 0

1054 0 0 0 0 0 0 0 0 0 0 0 0 0 0 0 0.89 0.11 0 0 0

1055 0 0 0 0 0 0 0 0 0 1 0 0 0 0 0 0 0 0 0 0

1056 0 0 0 0 0 0 0 0 0 1 0 0 0 0 0 0 0 0 0 0

1057 0 0 0 0 0 0 0 0 0 0 0 0.01 0 0 0 0.99 0 0 0 0

1058 0 0 0 0 0 0 0 0 0 0 0 1 0 0 0 0 0 0 0 0

1059 0 0 0 0 0 0 0 0 0 0 0 0 0 0 0 0 1 0 0 0

1060 0 0 1 0 0 0 0 0 0 0 0 0 0 0 0 0 0 0 0 0

1061 0 0 0 0 0 0 0 0 0 0 0 0 0 0 0 0 0 1 0 0

1062 0 0 0 0 0 0 0 0.02 0 0 0.9 0 0 0 0 0 0.08 0 0 0

1063 0 0 0 0 0 1 0 0 0 0 0 0 0 0 0 0 0 0 0 0

1064 0 0 0 0 0 0 0 0 0 1 0 0 0 0 0 0 0 0 0 0

1065 0 0 0 1 0 0 0 0 0 0 0 0 0 0 0 0 0 0 0 0

1066 0 0 0 1 0 0 0 0 0 0 0 0 0 0 0 0 0 0 0 0

1067 0 0 0 0 0 0 0 1 0 0 0 0 0 0 0 0 0 0 0 0

1068 0 0 1 0 0 0 0 0 0 0 0 0 0 0 0 0 0 0 0 0

1069 0 0 0 0 0 0 0 0 0 0 0 0 0 0 1 0 0 0 0 0

1070 1 0 0 0 0 0 0 0 0 0 0 0 0 0 0 0 0 0 0 0

- 0 0 0 0 0 0 0 0 0 0 0 0 0 0 0 0 0 0 0 0

1071 0 0 0 0 0 0 0 0 0 1 0 0 0 0 0 0 0 0 0 0

1072 0 0 0 0 0 1 0 0 0 0 0 0 0 0 0 0 0 0 0 0

1073 0 0 0 0 0 0 0 1 0 0 0 0 0 0 0 0 0 0 0 0

1074 0 0 0 0 0 0 0 0 0 0 0 0 1 0 0 0 0 0 0 0

1075 0 0 1 0 0 0 0 0 0 0 0 0 0 0 0 0 0 0 0 0

1076 0 0 0 0 0 0 0 0 0 1 0 0 0 0 0 0 0 0 0 0

1077 0 0 0 0 0 0 0 0 0 0 0 0 0 0 0 0 0 1 0 0

1078 0 0 0 0 0 0 0 0 0 0 0 0.01 0 0 0 0.99 0 0 0 0

1079 0 0 0 0 0 0 0 0 0 0 0 0 0 0.99 0 0 0 0 0 0

1080a 0 0 0 0 0 0 0 0 0 0 0 0 0 1 0 0 0 0 0 0

1080b 0.01 0 0 0 0 0.98 0 0 0 0 0 0 0 0 0.01 0.01 0 0 0 0

1081 0 0 0 0 0 0 0 0 0 0 0 0 0 1 0 0 0 0 0 0

>Node CID 1R

A C D E F G H I K L M N P Q R S T V W Y

1040 0 0 0 1 0 0 0 0 0 0 0 0 0 0 0 0 0 0 0 0

1041 0.3 0 0 0 0 0.13 0 0 0 0 0 0 0 0 0 0.53 0.03 0 0 0

1042 0 0 0 0 0 0 0 0 0 0 0 0 0 0.99 0.01 0 0 0 0 0

1043 0 0 0 0 0 0 0 0 0 0 0 0.98 0 0 0 0.02 0 0 0 0

1044 0 0 1 0 0 0 0 0 0 0 0 0 0 0 0 0 0 0 0 0

1045 0 0 0 1 0 0 0 0 0 0 0 0 0 0 0 0 0 0 0 0

1046 0 0 0 0 0 0 0 0 0.98 0 0 0 0 0 0.02 0 0 0 0 0

1047 1 0 0 0 0 0 0 0 0 0 0 0 0 0 0 0 0 0 0 0

1048 0 0 0 0 0 0 0 0 0 1 0 0 0 0 0 0 0 0 0 0

1049 0 0 0 0 0 0 0 0 0 1 0 0 0 0 0 0 0 0 0 0

1050 0 0 0.72 0.28 0 0 0 0 0 0 0 0 0 0 0 0 0 0 0 0

1051 0 0 0 0 0 0 0 0 0 0 0 0 0 1 0 0 0 0 0 0

1052 0 0 0 0 0 0 0 0 0 1 0 0 0 0 0 0 0 0 0 0

1053 0 0 1 0 0 0 0 0 0 0 0 0 0 0 0 0 0 0 0 0

1054 0 0 0 0 0 0 0 0 0 0 0 0 0 0 0 1 0 0 0 0

1055 0 0 0 0 0.02 0 0 0 0 0.98 0 0 0 0 0 0 0 0 0 0

1056 0 0 0 0 0 0 0 0 0 1 0 0 0 0 0 0 0 0 0 0

1057 0 0 0 0 0 0 0 0 0 0 0 0.04 0 0 0 0.96 0 0 0 0

1058 0 0 0 0 0 0.02 0 0 0 0 0 0.25 0 0 0 0.72 0 0 0 0

1059 0 0 0 0 0 0 0 0 0 0 0 0 0 0 0 0 0.99 0 0 0

1060 0 0 1 0 0 0 0 0 0 0 0 0 0 0 0 0 0 0 0 0

1061 0 0 0 1 0 0 0 0 0 0 0 0 0 0 0 0 0 0 0 0

1062 0 0 0 0 0 0 0 0.03 0 0.06 0.7 0 0 0.07 0 0 0.08 0.04 0 0

1063 0 0 0.05 0.94 0 0.01 0 0 0 0 0 0 0 0 0 0 0 0 0 0

1064 0 0 0 0 0 0 0 0 0 1 0 0 0 0 0 0 0 0 0 0

1065 0.99 0 0 0.01 0 0 0 0 0 0 0 0 0 0 0 0 0 0 0 0

1066 0 0 0 1 0 0 0 0 0 0 0 0 0 0 0 0 0 0 0 0

1067 0 0 0 0 0 0 0 0.98 0 0.02 0 0 0 0 0 0 0 0 0 0

1068 0 0 1 0 0 0 0 0 0 0 0 0 0 0 0 0 0 0 0 0

1069 0 0 0 0 0 0 0 0 0 0 0 0 0 0 1 0 0 0 0 0

1070 1 0 0 0 0 0 0 0 0 0 0 0 0 0 0 0 0 0 0 0

- 0 0 0 0 0 0 0 0 0 0 0 0 0 0 0 0 0 0 0 0

1071 0 0 0 0 0 0 0 0 0 1 0 0 0 0 0 0 0 0 0 0

1072 0 0 0 0 0 1 0 0 0 0 0 0 0 0 0 0 0 0 0 0

1073 0 0 0 0 0 0 0 1 0 0 0 0 0 0 0 0 0 0 0 0

1074 0 0 1 0 0 0 0 0 0 0 0 0 0 0 0 0 0 0 0 0

1075 0 0 0 0.01 0 0 0 0 0.99 0 0 0 0 0 0 0 0 0 0 0

1076 0 0 0 0 0 0 0 0 0 1 0 0 0 0 0 0 0 0 0 0

1077 0 0 0 0 0 0 0 0 0 0 0 0 0 0 0 0 0 1 0 0

1078 0.03 0.01 0.01 0.02 0 0.01 0.05 0 0.02 0.01 0 0.08 0.05 0.18 0.04 0.43 0.04 0 0 0

1079 0 0 0 0 0 0 0 0 0 0 0 0 0 0.99 0 0 0 0 0 0

1080a 0 0 0 0 0 0 0 0 0 0 0 0 0 1 0 0 0 0 0 0

1080b 0.05 0 0 0 0 0.61 0 0 0 0 0 0.01 0 0 0 0.32 0.01 0 0 0

1081 0 0 0 0 0 0.98 0 0 0 0 0 0 0 0.01 0 0 0 0 0 0

>Node CID 2R

A C D E F G H I K L M N P Q R S T V W Y

1040 0 0 0 1 0 0 0 0 0 0 0 0 0 0 0 0 0 0 0 0

1041 0.31 0 0 0 0 0.13 0 0 0 0 0 0 0 0 0 0.54 0.03 0 0 0

1042 0 0 0 0 0 0 0 0 0 0 0 0 0.02 0.98 0 0 0 0 0 0

1043 0 0 0 0 0 0 0 0 0 0 0 0.68 0 0 0 0.31 0 0 0 0

1044 0 0 1 0 0 0 0 0 0 0 0 0 0 0 0 0 0 0 0 0

1045 0 0 0 1 0 0 0 0 0 0 0 0 0 0 0 0 0 0 0 0

1046 0 0 0 0 0 0.01 0 0 0.72 0 0 0 0 0 0.27 0 0 0 0 0

1047 0.97 0 0 0 0 0 0 0 0 0 0 0 0 0 0 0 0.03 0 0 0

1048 0 0 0 0 0 0 0 0 0 1 0 0 0 0 0 0 0 0 0 0

1049 0 0 0 0 0 0 0 0 0 1 0 0 0 0 0 0 0 0 0 0

1050 0 0 0.99 0.01 0 0 0 0 0 0 0 0 0 0 0 0 0 0 0 0

1051 0 0 0 0 0 0 0 0 0 0 0 0 0 1 0 0 0 0 0 0

1052 0 0 0 0 0 0 0 0 0 1 0 0 0 0 0 0 0 0 0 0

1053 0 0 0.99 0 0 0 0 0 0 0 0 0 0 0 0 0 0 0 0 0

1054 0 0 0 0 0 0 0 0 0 0 0 0 0 0 0 0.99 0.01 0 0 0

1055 0.01 0 0 0 0.01 0 0 0 0 0.98 0 0 0 0 0 0 0 0 0 0

1056 0 0 0 0 0 0 0 0 0 1 0 0 0 0 0 0 0 0 0 0

1057 0 0 0 0 0 0 0 0 0.01 0 0 0.04 0 0 0 0.95 0 0 0 0

1058 0 0 0 0 0 0 0 0 0 0 0 0.99 0 0 0 0.01 0 0 0 0

1059 0.01 0 0 0 0 0 0 0.03 0 0 0 0 0 0 0 0.01 0.94 0 0 0

1060 0 0 1 0 0 0 0 0 0 0 0 0 0 0 0 0 0 0 0 0

1061 0 0 0 1 0 0 0 0 0 0 0 0 0 0 0 0 0 0 0 0

1062 0 0 0 0 0 0 0 0.02 0 0.08 0.85 0 0 0.01 0 0 0.03 0.01 0 0

1063 0 0 0 0.01 0 0.99 0 0 0 0 0 0 0 0 0 0 0 0 0 0

1064 0 0 0 0 0 0 0 0 0 1 0 0 0 0 0 0 0 0 0 0

1065 0 0 0 0.99 0 0 0 0 0 0 0 0 0 0 0 0 0 0 0 0

1066 0 0 0 1 0 0 0 0 0 0 0 0 0 0 0 0 0 0 0 0

1067 0 0 0 0 0 0 0 1 0 0 0 0 0 0 0 0 0 0 0 0

1068 0 0 1 0 0 0 0 0 0 0 0 0 0 0 0 0 0 0 0 0

1069 0 0 0 0 0 0 0 0 0 0 0 0 0 0 1 0 0 0 0 0

1070 1 0 0 0 0 0 0 0 0 0 0 0 0 0 0 0 0 0 0 0

- 0 0 0 0 0 0 0 0 0 0 0 0 0 0 0 0 0 0 0 0

1071 0 0 0 0 0 0 0 0 0 1 0 0 0 0 0 0 0 0 0 0

1072 0 0 0 0 0 1 0 0 0 0 0 0 0 0 0 0 0 0 0 0

1073 0 0 0 0 0 0 0 1 0 0 0 0 0 0 0 0 0 0 0 0

1074 0 0 0 0 0 0 0 0 0 0 0 0 1 0 0 0 0 0 0 0

1075 0 0 0.18 0.78 0 0 0 0 0.03 0 0 0 0 0 0 0 0 0 0 0

1076 0 0 0 0 0 0 0 0 0 1 0 0 0 0 0 0 0 0 0 0

1077 0 0 0 0 0 0 0 0 0 0 0 0 0 0 0 0 0 1 0 0

1078 0 0 0 0 0 0 0 0 0 0 0 0.05 0 0 0 0.92 0.01 0 0 0

1079 0 0 0 0 0 0 0 0 0 0 0 0 0 1 0 0 0 0 0 0

1080a 0 0 0 0 0 0 0 0 0 0 0 0 0 1 0 0 0 0 0 0

1080b 0.04 0 0 0 0 0.67 0 0 0 0 0 0 0 0 0 0.27 0.01 0 0 0

1081 0 0 0 0 0 0 0 0 0 0 0 0 0 0.99 0 0 0 0 0 0
